# Non-HDL-C/HDL-C Ratio and N-Terminal Pro-B-Type Natriuretic Peptide in the general and hospital-based population: a cross-sectional validation study

**DOI:** 10.3389/fendo.2025.1739961

**Published:** 2026-01-09

**Authors:** Haitao Xie, Jianghong Li, Le Shen, Peng Yu, Shi Wang, Xiaohu Chen, Shuhua Tang

**Affiliations:** 1 Affiliated Hospital of Nanjing University of Chinese Medicine, Nanjing, China; 2 Department of Cardiology, Jiangsu Province Hospital of Chinese Medicine, Nanjing, China; 3 Nanjing University of Chinese Medicine, Nanjing, China

**Keywords:** elevated NT-proBNP, general population, hospital-based, negative association, NHHR

## Abstract

**Background:**

As an innovative composite lipid assessment index, the Non-HDL-C/HDL-C Ratio (NHHR) offers a more holistic appraisal of lipid metabolic profile. This investigation seeks to elucidate the association between NHHR and elevated N-Terminal Pro-B-Type Natriuretic Peptide (NT-proBNP) levels in the general and hospital-based population.

**Methods:**

The research participants were drawn from the National Health and Nutrition Examination Survey (NHANES, 1999-2004, Group 1) and from hospital inpatients within the Cardiology Department of Jiangsu Province Traditional Chinese Medicine Hospital (JSHTCM, January to June 2025, Group 2). We preliminarily evaluated and adjusted for the original prevalence and age-standardized prevalence of NT-proBNP elevation in different populations. A stepwise inference generalized linear modeling approach was employed to explore the association between NHHR and NT-proBNP elevation, with a restrictive cubic spline (RCS) used to visualize the simulations, evaluated correlation trends through trend tests, observed inter-group differences through subgroup analyses, assessed overall population results robustness through interaction tests. Additionally, based on the non-linear associations, we further refined the analysis through threshold and saturation effects, as well as piecewise regression analysis.

**Results:**

In Group 1, females exhibited a higher age-standardized prevalence of NT-proBNP elevation than males (20.51% vs. 10.74%), which declined progressively as NHHR levels increased. Generalized linear models demonstrated a notable inverse link between NHHR and NT-proBNP elevation in both groups, and the results of the RCS plots are largely consistent with the findings from the generalized linear models. Additionally, further threshold and saturation effects analysis identified 4.3 as the NHHR inflection point in Group 1. Piecewise regression analysis indicated that below this threshold, higher NHHR corresponded to a reduced likelihood of NT-proBNP elevation. Specifically, for each standard unit increase in NHHR, the probability of elevated NT-proBNP decreased by 24% [HR (95% CI): 0.76 (0.68, 0.86), *P* < 0.001]. When NHHR exceeded the inflection point, the association between the two variables no longer remained significant.

**Conclusions:**

In the general and hospital-based population, a negative correlation was identified between the increase in NHHR and NT-proBNP elevation levels, corroborating the utility of NHHR as a simple composite lipid index for identifying changes in NT-proBNP.

## Introduction

In the past few years, there has been a notable increase in the occurrence of cardiovascular diseases (CVD), making it one of the key health challenges worldwide. As the population continues to age and the effects of unhealthy lifestyles remain, it is anticipated that the rates of morbidity and mortality linked to CVD will further escalate, presenting a significant obstacle for public health systems around the globe (1, 2).

It is well-established that dyslipidemia constitutes a major risk factor for CVD. In populations at elevated CVD risk, the dyslipidemic profile is characterized specifically by reduced high-density lipoprotein cholesterol (HDL-C) levels accompanied by elevated non-HDL-C levels. The latter encompasses atherogenic lipoproteins, including apolipoprotein B (ApoB)-carrying low-density lipoprotein cholesterol (LDL-C), triglyceride-rich lipoproteins (TRLs) and their remnants, and lipoprotein(a) (3, 4). While traditional risk assessment often focuses on single metrics such as LDL-C, a broader evaluation of residual lipid risk represented by non-HDL-C provides a more comprehensive assessment of cardiovascular risk. Notably, clinical guidelines have increasingly emphasized the importance of non-HDL-C. For instance, reports from the American Diabetes Association have recommended incorporating non-HDL-C into management targets for diabetes and its complications (5), and the UK National Institute for Health and Care Excellence (NICE) has suggested that non-HDL-C could serve as a primary target for reducing CVD risk, superseding LDL-C in certain contexts (6). Substantial evidence from cohort studies further supports the superior predictive value of non-HDL-C for CVD outcomes (7, 8). Building upon this rationale, the non-HDL-C to HDL-C ratio (NHHR) has emerged as a composite lipid index that integrates both atherogenic (non-HDL-C) and protective (HDL-C) components, thereby reflecting the balance between pro-atherogenic lipid burden (9) and anti-atherogenic capacity. Consequently, NHHR demonstrates enhanced predictive accuracy and diagnostic relevance in evaluating conditions such as atherosclerosis, myocardial infarction, and insulin resistance (10–14).

NT-proBNP is a validated cardiac biomarker indicative of myocardial performance, secreted by ventricular cardiomyocytes in response to hemodynamic stress from increased intracardiac pressure or volume overload (15, 16). It acts as a marker for both cardiac stress and myocardial damage, whether acute or chronic. To date, the association between the NHHR and circulating NT-proBNP concentrations has not been comprehensively investigated. Therefore, this investigation consolidates data derived from two epidemiological groups: NHANES and a hospital-based validation group, investigating the relationship between NHHR and NT-proBNP levels among the general and hospital-based population, aiming to offer new clinical insights.

## Methods

### Study participants

In this cross-sectional validation study, we recruited two independent groups drawn from distinct sources. Group 1 consisted of adults (≥20 years) from NHANES cycles conducted between 1999 and 2004. Group 2 consisted of inpatients admitted to the Cardiology Department of the JSHTCM from January to June 2025, all patients underwent comprehensive examinations after admission, including standard electrocardiogram, echocardiography, coronary CTA or coronary angiography. Within the context of this research, all participants were drawn from the respective populations and had no prior history of overt CVD. Exclusion criteria were as follows: Group 1: 1. a history of physician-diagnosed CVD; 2. missing data on the cardiac biomarkers of interest; 3. presence of severe renal impairment; 4. incomplete data for essential covariates. Group 2: participants were excluded based on the presence of any of the following clinical conditions:1. angiographically or CTA confirmed significant coronary artery stenosis (>50% luminal narrowing in any major epicardial vessel); 2. depressed left ventricular systolic function (ejection fraction <40%); 3. documented tachyarrhythmias (e.g., atrial fibrillation, atrial flutter). 4. unavailability of complete clinical or laboratory data required for analysis.

**Figure 1:**
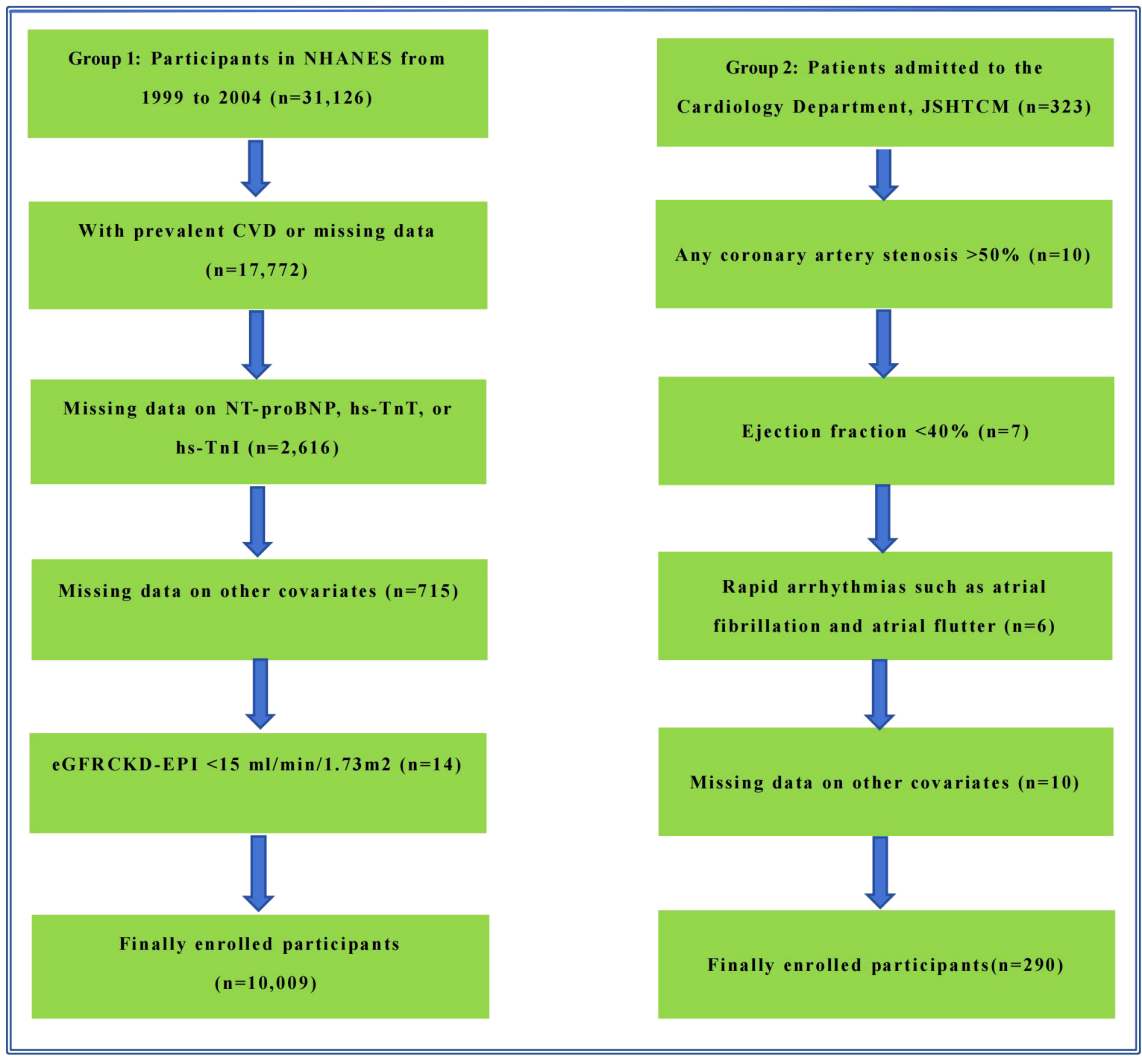
Flowchart delineating study methodology and exclusion criteria.

Regarding the specific research design, we employed a two-stage analytical approach. First, we conducted an exploratory analysis using the nationally representative data (Group 1) to investigate whether an association exists between NHHR and NT-proBNP levels. Subsequently, we performed an independent validation of these initial findings using the hospital-sourced data (Group 2) to strengthen the robustness and credibility of our conclusions. Following rigorous screening of all individuals, the final analytical cohorts included 10,009 (Group 1) and 290 (Group 2) adult individuals free of CVD. The detailed study protocol and exclusion parameters are delineated in the flowchart (**Figure 1**).

### Evaluation of the NHHR

The exposure variable chosen for this study is NHHR, referring to current relevant research, the precise computational formula for NHHR is as follows: NHHR = [(TC (mmol/L) - HDL-C (mmol/L))/HDL-C (mmol/L)] (17, 18).

### Measurement of NT-proBNP

In Group 1, fasting serum specimens were obtained and stored at the University of Maryland School of Medicine. These specimens underwent comprehensive quantification by trained analysts from Johns Hopkins University. Primary data acquisition involved quantification of NT-proBNP concentrations utilizing the Roche Cobas e601 automatic analyzer, with a detection range of 5 pg/mL to 35,000 pg/mL. Conversely, in Group 2, serum NT-proBNP levels were assessed using automated analyzers from the Laboratory of Diagnostics, JSHTCM, which exhibited a lower analytical limit of detection at 20 pg/mL.

### Assessment of elevated NT-proBNP

Based on the NT-proBNP concentrations across the cohort, the overall population was classified in accordance with the established risk stratification ranges for cardiac biomarkers and with reference to findings from other studies. In the analysis, an elevated level of NT-proBNP was determined to be any measurement that equaled or exceeded 125 pg/mL (19–21).

### Covariate definitions

All participants across the two groups underwent comprehensive data collection, including sociodemographic variables, behavioral lifestyle parameters, and clinical health metrics, with standardized recording of basic characteristics including age, gender, and BMI. The smoking status of each participant was evaluated and classified utilizing a systematically designed questionnaire. Clinical health metrics include fasting serum biochemical indicators (urea, creatinine, triglycerides, total cholesterol, HDL-C, fasting blood glucose, and HbA1c). Diabetes was operationally defined in three ways: individuals either reported a personal history of diabetes that had been diagnosed by a physician, exhibited an HbA1c level of 6.5% or greater, or were currently using medications prescribed for diabetes management. Individual blood pressure levels were measured following the standardized process, with participants having their right upper arm blood pressure measured three times consecutively. Hypertension was defined by clinical criteria including a mean systolic blood pressure of ≥140 mmHg, a diastolic blood pressure of ≥90 mmHg, self-reported physician diagnosis of hypertension, or current pharmacological management with antihypertensive agents. Renal function evaluation was conducted employing the Chronic Kidney Disease Epidemiology Collaboration (CKD-EPI) formula, with patients exhibiting advanced renal impairment (eGFR ≤ 15 ml/min/1.73 m^2^) excluded from the dataset. Medication use was extracted from prescription records within the most recent month, with a primary focus on lipid-lowering medications.

### Statistical analysis

By leveraging the continuous range of NHHR values, we partitioned the two groups into four separate categories (Q1–Q4) to characterize baseline participant characteristics, including sociodemographic factors and cardiovascular risks, and to examine differences across these groups. Given the oversampling of certain age groups in NHANES and the resulting age distribution disparities, for Group 1, the age-standardized prevalence of elevated NT-proBNP was determined. This calculation employed the 2000 U.S. population age distribution as the standard for each group.

To explore the connection between NHHR and elevated NT-proBNP in the general and hospital-based population, we constructed various weighted generalized linear models for stepwise inference. NHHR was treated as both continuous and categorical variables, with the categorical data segmented into quartiles, where Q1 was taken as the reference. The models were characterized as follows respectively: Group 1: crude (unadjusted) model. Model 1: incorporated covariates including gender, age, race, and education level. Model 2: further refined the analysis by adjusting for urea, creatinine (Cr), triglycerides (TG), uric acid (Ua), fasting blood glucose (FBG), HbA1c, hs-TnT, hs-TnI, eGFR, BMI, hypertension, diabetes, and smoking. Group 2: the crude model remained unadjusted; Model 1 included covariates for gender and age; Model 2 further extended Model 1 by adding variables such as urea, creatinine, triglycerides, uric acid, fasting blood glucose, HbA1c, eGFR, BMI, hypertension, diabetes, smoking, and medication use. Trend analyses were conducted to assess the robustness and consistency of the research outcomes.

In two groups, we examined whether the relationship between NHHR and elevated NT-proBNP follows a nonlinear pattern by employing visualization-based modeling with restricted cubic splines. For this approach, four knots were placed at the 5th, 35th, 65th, and 95th percentiles, consistent with the adjustments used in Model 2. Building upon this, within Group 1, we employed segmented regression to fit each interval independently. By comparing segmented regression to overall regression and utilizing the log-likelihood ratio test to statistically ascertain the presence of threshold and saturation effects. Finally, the precise inflection point was determined via a recursive two-step estimation procedure. Furthermore, robustness checks were conducted through subgroup analyses stratified by gender, age (20-39y, 40-59y, 60+y), BMI (normal, overweight, obese), hypertension, and diabetes, interaction tests were conducted to determine whether the overall findings varied across subgroups.

In Group 1, according to the prescribed analytical protocols, all statistical evaluations integrated the survey sampling weights representative of the study period to ensure accurate population inference. The specific calculation for the survey weights employed a weighted combination: 2/3 × wtint4yr (1999-2002) + 1/3 × wtint2yr (2003-2004). Data analyses were executed using R statistical software (version 4.4.0; http://www.r-project.org). Statistical significance was defined as a two-tailed P-value less than 0.05.

## Results

### Baseline characteristics of participants

In Group 1, a total of 10,009 participants of U.S. general population were included. Based on NHHR quartiles, baseline characteristics across groups are detailed in **Table 1**. Participants in the highest NHHR quartile (Q4), relative to those in the lowest quartile (Q1), exhibited distinct phenotypic and metabolic profiles. Specifically, the group with elevated NHHR levels tended to be predominantly male, were generally older in age, displayed a lower level of educational attainment, and were chiefly Non-Hispanic White. Additionally, this group exhibited higher measurements of BMI, along with increased serum levels of urea, Cr, UA, TG, TC, FBG, and HbA1c. Furthermore, the high NHHR group presented a heightened burden of cardiovascular risk factors—such as hypertension, diabetes, and smoking. Notably, as NHHR levels increased, cardiac biomarkers such as hs-TnT and hs-TnI rose progressively, while NT-proBNP levels showed a gradual decline (**Table 1**). These differences among groups were statistically significant. Additionally, aside from age and the prevalence of hypertension, the other baseline characteristics in Group 2 were largely concordant with those observed in Group 1 (**Supplementary Table S1**).

**Table 1:** Characteristics of the general population stratified by NHHR status within Group 1.

NHHR status	Q1	Q2	Q3	Q4	*p*
n	2507	2501	2499	2502	
	< 2.099	2.099 - < 2.890	2.890 - < 3.913	≥ 3.913	
Age	42.35 (16.24)	43.68 (16.57)	45.95 (16.13)	45.65 (14.51)	<0.001
Gender (%)					<0.001
Male	28.23	40.42	52.88	66.61	
Female	71.77	59.58	47.12	33.39	
Race (%)					<0.001
Mexican American	6.7	7.6	8.4	7.7	
Other Hispanic	4.2	6.3	6.5	6.6	
Non-Hispanic White	71.9	70.6	70.9	74.9	
Non-Hispanic Black	12.8	10.8	9.4	6.3	
Other Race	4.4	4.6	4.7	4.5	
Education (%)					<0.001
Less Than High School	15.8	17.6	19.2	21.2	
High School Diploma	22.4	25	25.9	29.7	
More Than High School	61.8	57.4	54.9	49.1	
Hypertension (%)					0.001
No	83.83	83.01	80.37	79.62	
Yes	16.17	16.99	19.63	20.38	
smoke (%)					<0.001
Never	56.54	53.42	50.87	44.52	
Current	20.9	23.45	23.82	29.84	
Former	22.56	23.13	25.31	25.64	
BMI (kg/m^2^)	25.25 (5.64)	27.43 (6.22)	29.12 (6.17)	30.21 (5.61)	<0.001
Urea (mmol/l)	4.41 (1.57)	4.63 (1.65)	4.77 (1.70)	4.82 (1.63)	<0.001
Creatinine (umol/l)	69.51 (19.99)	71.39 (23.64)	75.15 (22.05)	77.17 (23.40)	<0.001
TC (mmol/l)	4.63 (0.87)	4.98 (0.88)	5.34 (0.89)	5.98 (1.08)	<0.001
HDL (mmol/l)	1.79 (0.40)	1.43 (0.26)	1.23 (0.21)	0.99 (0.19)	<0.001
TG (mg/dl)	79.02 (38.52)	102.99 (51.09)	136.89 (72.78)	228.21 (191.14)	<0.001
Uric acid (umol/l)	275.73 (73.73)	302.44 (79.94)	327.75 (78.13)	351.97 (81.97)	<0.001
FBG (mg/dl)	89.09 (20.82)	91.19 (21.25)	93.53 (24.02)	98.78 (34.06)	<0.001
HbA1c (%)	5.23 (0.61)	5.33 (0.67)	5.46 (0.80)	5.62 (1.06)	<0.001
NT-proBNP, pg/mL	103.63 (382.09)	102.00 (465.41)	79.88 (234.43)	69.83 (217.60)	<0.001
Elevated NT-proBNP (%)					<0.001
No	81.46	83.5	86.76	89.63	
Yes	18.54	16.5	13.24	10.37	
Hs-troponin T, ng/L	5.50 (4.88)	6.10 (7.56)	6.57 (6.64)	6.55 (7.20)	<0.001
Hs-troponin I, ng/L	2.03 (4.96)	2.53 (13.18)	2.87 (10.27)	3.10 (16.42)	<0.001
Diabetes (%)					<0.001
No	96.87	95.44	93.89	92.24	
Yes	3.13	4.56	6.11	7.76	

### Prevalence of NT-proBNP elevation

**Figure 2:**
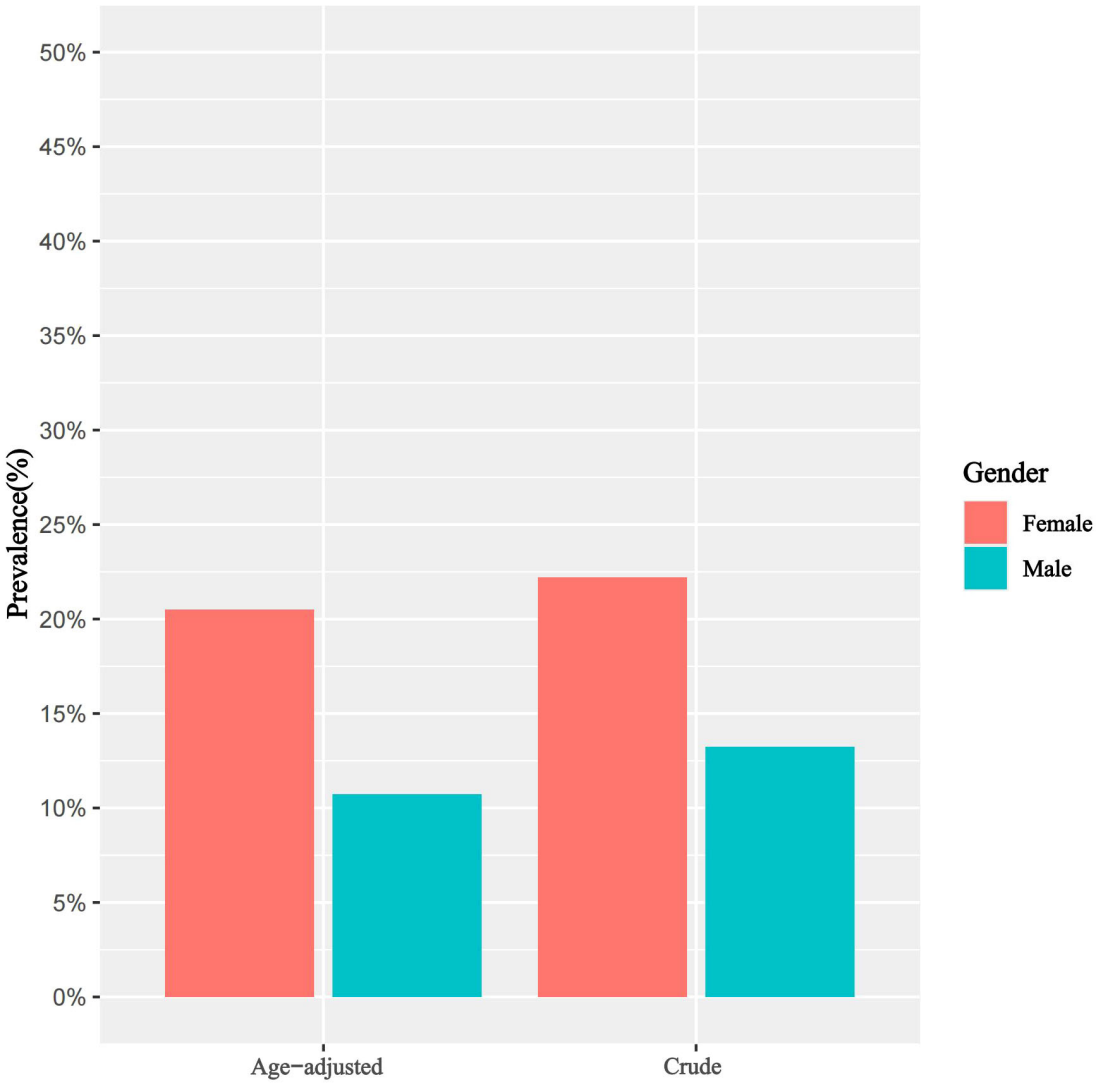
Crude and age-standardized prevalence of NT-proBNP elevation in the general population, stratified by gender, Group 1.

In Group 1, females demonstrated a higher crude prevalence of NT-proBNP elevation compared to males (females vs. males: 22.19% vs. 13.23%). Additionally, the prevalence exhibits a decreasing trend with increasing NHHR stratifications. Upon age-standardization, the prevalence was adjusted to 20.51% in females and 10.74% in males, as detailed in **Table 2** and illustrated in **Figure 2**.

**Table 2:** Crude and age-standardized prevalence of NT-proBNP elevation in the general population, stratified by gender, Group 1.

NT-proBNP elevation	Male	Female
Crude prevalence	13.23%	22.19%
Age-adjusted prevalence	10.74%	20.51%

### Correlations between NHHR and NT-proBNP elevation

**Figure 3:**
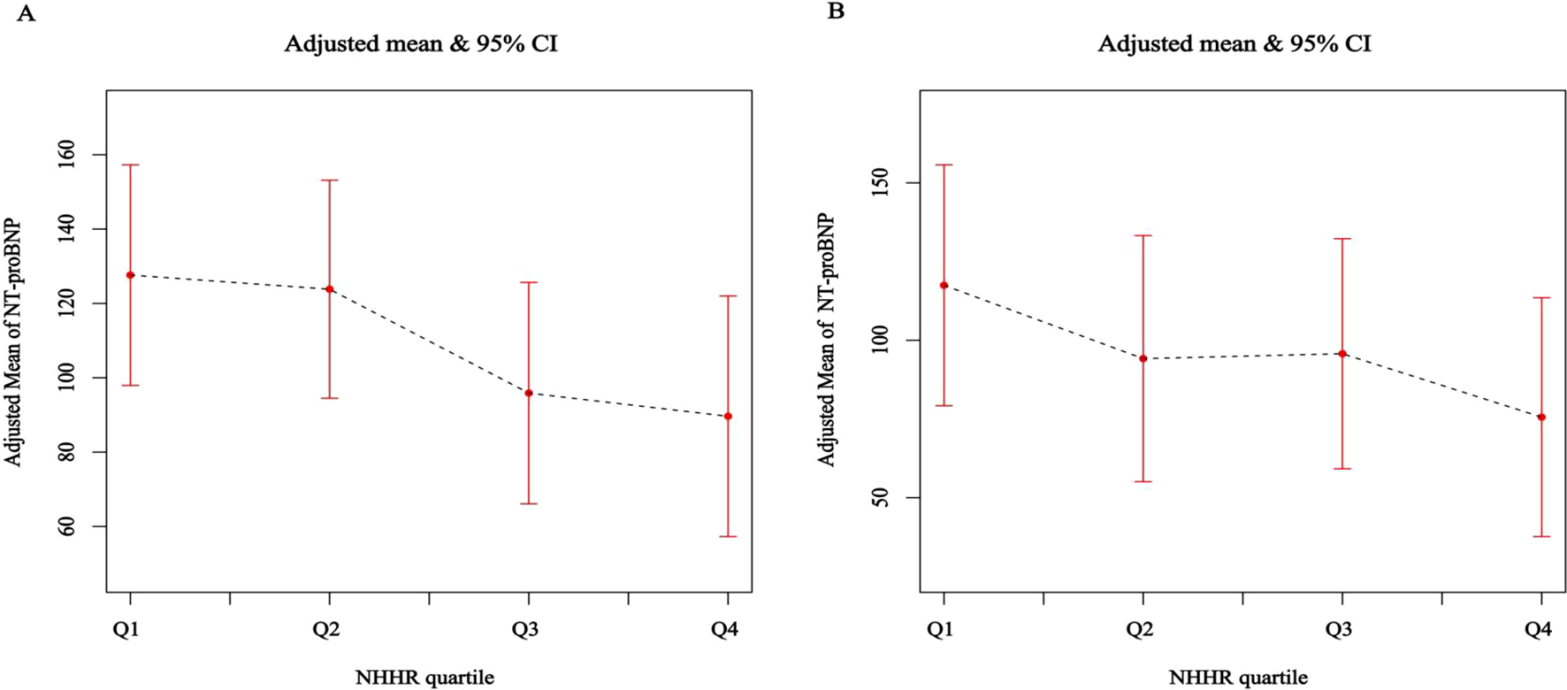
**(A, B)** Adjusted association of NHHR (quartile) with serum NT-proBNP in the general and hospital-based population.

In Group 1, utilizing a weighted generalized linear model and accounting for variables such as gender, age, race, and education, a notable inverse link emerged between continuous NHHR and elevated NT-proBNP. Specifically, higher NHHR values correlated with lower serum levels of NT-proBNP. This negative association remained robust and statistically significant even with further adjustment for additional covariates (model 2); quantitatively, each standard unit increase in NHHR corresponded to about a 6.83 pg/mL decline in serum NT-proBNP [β (95% CI): -6.83 (-12.08, -1.59), *P* for trend <0.001], and the odds of NT-proBNP elevation decreased by 16% [OR (95% CI): 0.84 (0.76, 0.92), *P* = 0.001]. Additionally, NHHR quartile analysis showed that individuals in the top quartile (Q4) had a 47% lower probability of NT-proBNP elevation per standard unit increase in NHHR when compared with those in the bottom quartile (Q1) [OR (95% CI): 0.53 (0.37, 0.78), *P* for trend < 0.001] (**Table 3**; **Figure 3A**).

**Table 3:** Adjusted association of NHHR with NT-proBNP elevation in general population, Group 1.

Association of NHHR with NT-proBNP elevation, Group 1						
Outcome	Crude model		Model I		Model II	
	OR (95% CI)	*p* value	OR (95% CI)	*p* value	OR (95% CI)	*p* value
NHHR	0.83 (0.79, 0.88)	< 0.001	0.83 (0.77, 0.89)	< 0.001	0.84 (0.76, 0.92)	0.001
NHHR (quartile)						
Q1	Reference	< 0.001	Reference	< 0.001	Reference	0.018
Q2	0.87 (0.71, 1.05)	0.137	0.81 (0.64, 1.01)	0.066	0.83 (0.64, 1.08)	0.164
Q3	0.67 (0.56, 0.80)	< 0.001	0.54 (0.44, 0.66)	< 0.001	0.55 (0.44, 0.69)	< 0.001
Q4	0.51 (0.41, 0.63)	< 0.001	0.50 (0.37, 0.66)	< 0.001	0.53 (0.37, 0.78)	0.002
P for trend		< 0.001		< 0.001		< 0.001
Association of NHHR with NT-proBNP elevation, Group 1						
Outcome	Crude model		Model I		Model II	
	β (95% CI)	*p* value	β (95% CI)	*p* value	β (95% CI)	*p* value
NHHR	-8.89 (-11.60, -6.19)	< 0.001	-10.15 (-13.27, -7.02)	< 0.001	-6.83 (-12.08, -1.59)	0.018
NHHR (quartile)						
Q1	Reference	< 0.001	Reference	< 0.001	Reference	0.002
Q2	-2.17 (-14.24, 9.91)	0.727	-5.42 (-15.50, 6.66)	0.386	-3.96 (-16.45, 8.53)	0.542
Q3	-23.79 (-35.76, -11.83)	< 0.001	-34.61 (-47.32, -21.90)	< 0.001	-23.52 (-34.95, -12.09)	< 0.001
Q4	-33.94 (-45.35, -22.53)	< 0.001	-41.56 (-55.02, -28.10)	< 0.001	-23.53 (-37.95, -9.11)	0.005
*P* for trend		< 0.001		< 0.001		< 0.001

The findings from Group 2 similarly demonstrate that as NHHR values increase, serum NT-proBNP concentrations exhibit a gradual decline among the general population, consistent with the generalized linear model results from Group 1. Concretely, each standard unit rise in NHHR corresponded to an approximate decrease of 21.32 pg/mL in serum NT-proBNP levels [β (95% CI): -21.32 (-36.92, -5.73), *P* for trend = 0.026], alongside a 55% reduction in the probability for elevated serum NT-proBNP levels [OR (95% CI): 0.45 (0.25, 0.77), *P* for trend = 0.035]. Furthermore, the decline in serum NT-proBNP levels observed in the highest quartile (Q4) relative to the lowest quartile (Q1) was notably more substantial (**Supplementary Table S2**; **Figure 3B**). Given that NT-proBNP is an established cardiac biomarker, this inverse association suggests that a more atherogenic lipid profile (reflected by a higher NHHR) may linked to lower levels of this cardiac marker in individuals without prevalent CVD.

### Restricted cubic splines

**Figure 4:**
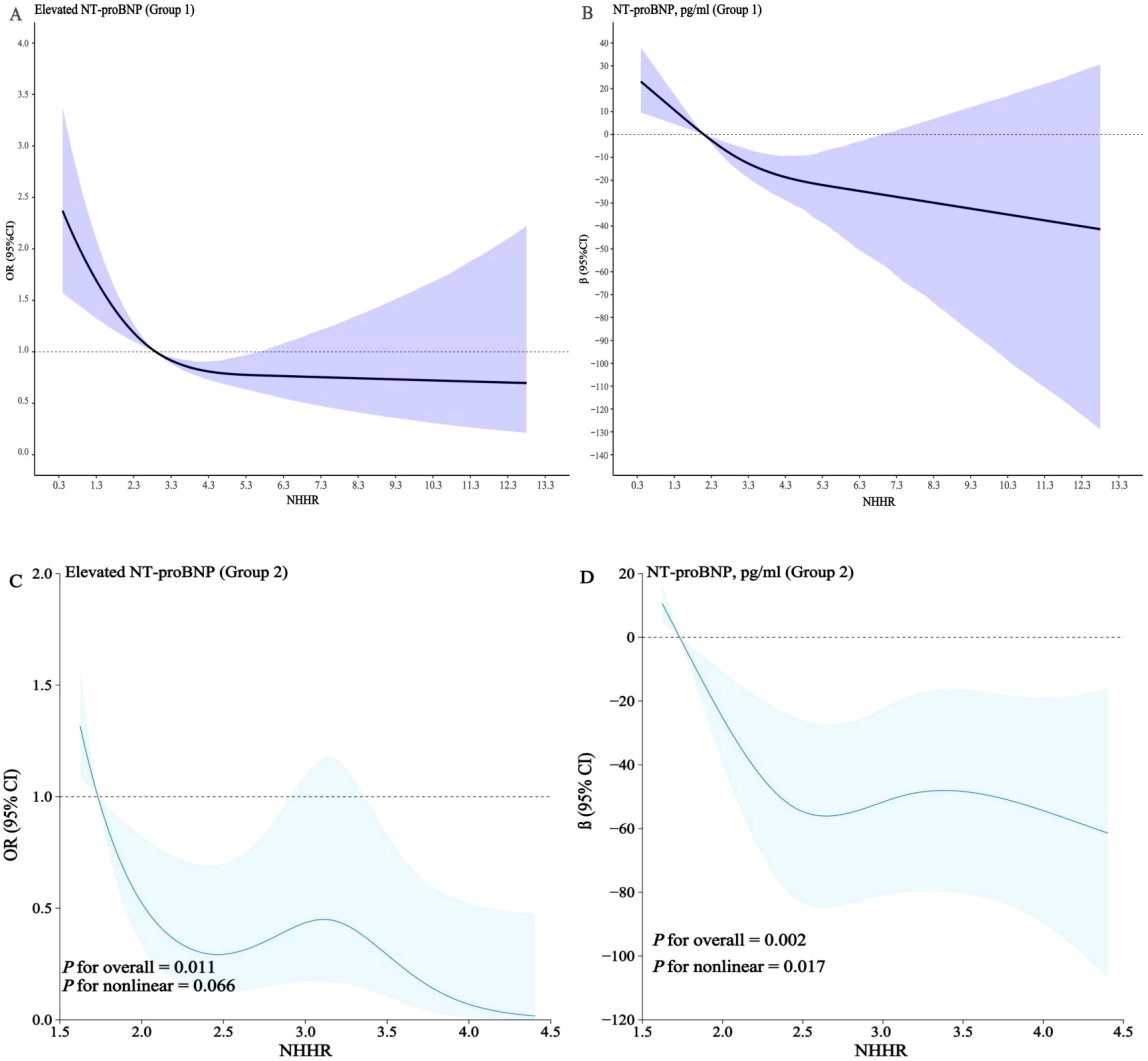
**(A–D)** RCS models illustrating the correlation between NHHR and serum NT-proBNP levels in the general and hospital-based population.

In two groups, we applied RCS modeling to visualize the association between NHHR and elevated NT-proBNP. Notably, we identified a significant nonlinear connection between these variables [*P* for nonlinear <0.001; *P* for nonlinear =0.017] (**Figure 4**). Subsequently, we conducted further analyses in Group 1 to determine the threshold and saturation effects, revealing that the inflection point for the associations between NHHR and elevated NT-proBNP was 4.3 (likelihood ratio test *P* = 0.001). To further investigate this association, we employed a piecewise regression model using the entire study population. Results demonstrated an inverse correlation between NHHR and NT-proBNP concentrations below the identified inflection point, with each standard unit increase in NHHR associated with a 24% decreased probability of elevated NT-proBNP [OR (95% CI): 0.76 (0.68–0.86), *P* < 0.001]. Beyond the inflection threshold, the association was no longer statistically significant (**Table 4**).

**Table 4:** Piecewise regression between NHHR and NT-proBNP elevation in general population, Group 1.

Outcome	Model II	*P* value
	OR (95% CI)	
NHHR < 4.3	0.76 (0.68, 0.86)	< 0.001
NHHR ≥ 4.3	0.95 (0.74, 1.21)	0.649

Model II was adjusted for age, gender, race,education, urea, creatinine, triglycerides, uric acid, fasting blood glucose, HbA1c, hs-TnT, hs-TnI, eGFR, BMI, hypertension, diabetes, and smoking status.

### Subgroup analysis

**Figure 5:**
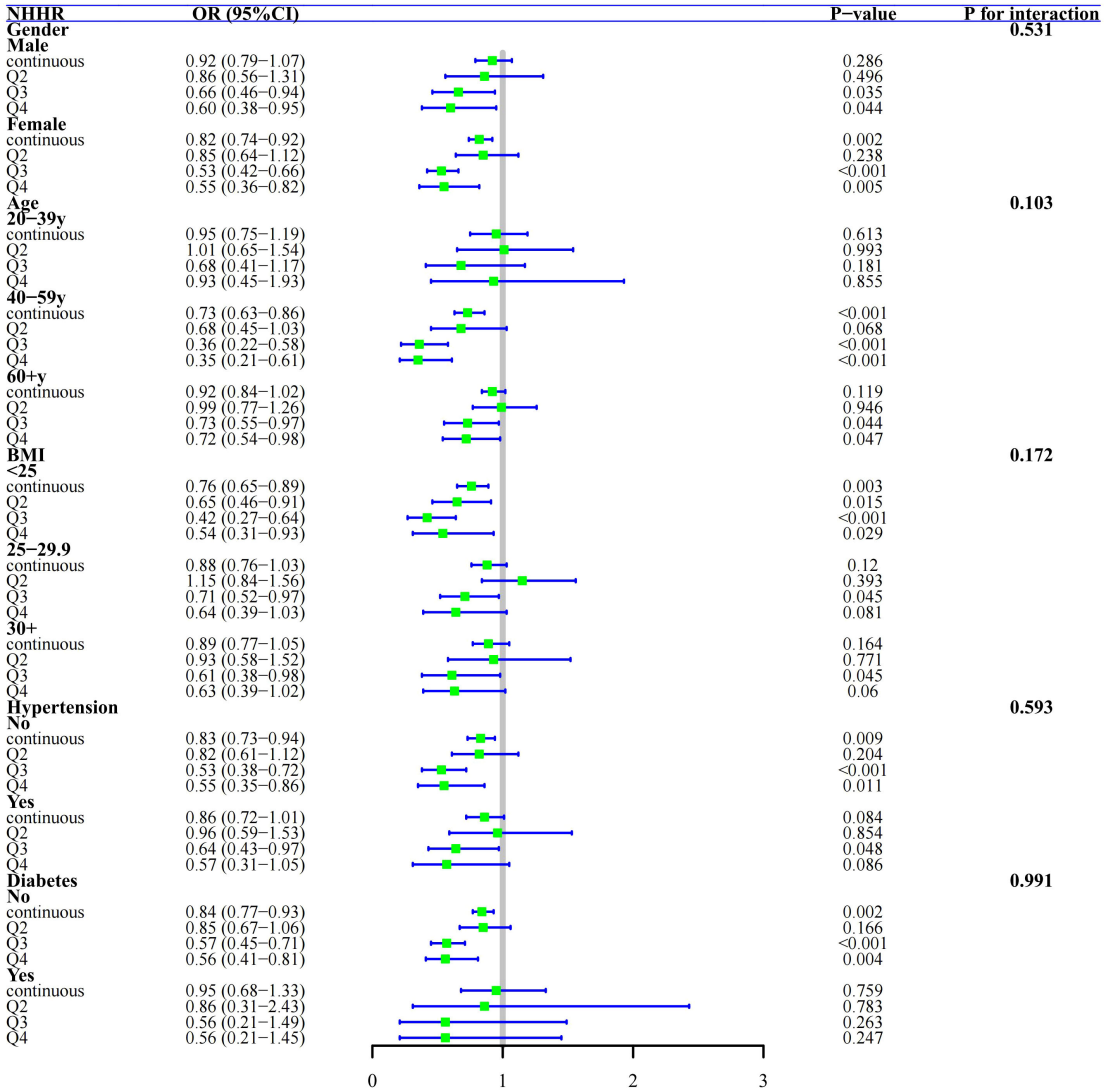
Subgroup Analysis and Forest Plots of NHHR with NT-proBNP elevation by gender, age, BMI, hypertension, and diabetes in general population, Group 1.

To explore the consistency of the link between NHHR and elevated NT-proBNP in Group 1, we stratified based on covariates such as gender, age, BMI, hypertension, and diabetes for subgroup analysis, which are considered potential modifying factors. Our findings indicate that participants with higher NHHR quartiles (Q4) have a notably reduced trend of increased NT-proBNP compared to those with lower NHHR levels (Q1) across various subgroups. Moreover, this negative correlation remains stable even when NHHR is in a continuous form. Interaction tests revealed no significant moderating effects of gender, age, BMI, hypertension, or diabetes on the association between NHHR and elevated NT-proBNP (**Figure 5**, all *P*-values for interaction > 0.05).

## Discussion

As an emerging lipid ratio index, NHHR serves to provide a thorough representation of lipid-related information pertinent to atherosclerosis and its prevention, thereby offering an enhanced evaluation of a person’s lipid health status. Existing studies have demonstrated that NHHR possesses significant clinical predictive value in various lipid-related diseases (22–24), and is notably associated with coronary artery disease, acute coronary syndrome, and major adverse cardiovascular events (25–27). Furthermore, lipid profiles offers critical insights into systemic lipid metabolism and overall cardiometabolic health, essential for CVD assessment (28). Additionally, A study by Yu et al. (29) revealed NHHR displays U-shaped and L-shaped associations with risks of all-cause mortality and cardiovascular mortality, respectively. When NHHR is below the inflection point, the association is negative; conversely, above the inflection point, it becomes positive. This further highlights the clinical predictive and disease management value of NHHR.

NT-proBNP, as a typical cardiac biomarker, demonstrates stable predictive capabilities in conditions such as acute myocardial injury and heart failure. Notably, various studies have indicated that NT-proBNP can also assist in identifying CVD risk factors in adult populations, including conditions such as obesity and metabolic disorders. In the Framingham study, plasma NT-proBNP levels inversely correlated with diverse features of metabolic syndrome, excluding hypertension, in individuals without a heart failure history (30). Moreover, in patients experiencing metabolic syndrome, NT-proBNP levels displayed a negative relationship with metabolic indicators, including serum cholesterol and TG (19, 31, 32). A multiethnic investigation into atherosclerosis revealed a nonlinear relationship: NT-proBNP levels below a specific inflection point exhibited inverse correlations with TC, LDL-C, and TG, while positively associating with HDL-C; surpassing this inflection point, these correlations lacked significance (33). In our research, we noted a negative relationship between the NT-proBNP and the NHHR, aligning with previous findings. Additionally, unlike studies focusing on individuals at high cardiovascular risk, our analysis centered on the general and hospital-based population without a history of CVD, this constitutes a notable strength of our investigation.

However, the observation of a higher NHHR (reflecting a greater atherosclerotic lipid burden) being associated with lower NT-proBNP levels in a CVD-free population appears to contradict the traditional role of NT-proBNP as a marker of cardiac stress and adverse prognosis. From a physiological perspective, beyond their established functions in counteracting the renin-angiotensin-aldosterone and sympathetic systems to regulate blood pressure and volume homeostasis, natriuretic peptides have also been shown to possess adipomodulatory properties, exerting metabolic effects by enhancing lipolysis, thermogenesis, and insulin sensitivity (34). Consequently, the observed inverse association may be plausible in populations with obesity or metabolic dysfunction. On a biological mechanism level, the connection between NHHR and NT-proBNP can be elucidated from the following perspectives. Firstly, BNP has a lytic effect in adipose tissue (35, 36) and dyslipidemia may lead to vascular endothelial damage, increasing the release of neutral endopeptidases, thereby accelerating BNP degradation. NT-proBNP and BNP are derived from different amino acid sites formed by the deglycosylation of pro-BNP1–108 via serine protease cleavage (37, 38). NT-proBNP may share similar physiological mechanisms within adipose tissue. Moreover, NT-proBNP has potential involvement in the metabolism of glucose and lipids (39). Insulin resistance, overexpression of cytokines in adipose tissue, and disturbances in the homeostasis of neurohormones related to obesity are also among the mechanisms contributing to this association (40, 41).

Nevertheless, as a cross-sectional analysis, it must be acknowledged that the inverse association observed between atherosclerotic lipid burden and NT-proBNP levels presents a notable departure from conventional understanding. Given the inability to establish causality, an explanation based solely on metabolic pathways remains incomplete. It is important to consider the potential for bidirectional regulatory interplay between this endogenous peptide system and overall metabolic homeostasis. For instance, reduced natriuretic peptide levels may compromise their metabolic protective effects, thereby exacerbating metabolic dysregulation and contributing to disordered lipid profiles (42). Conversely, in the context of adipose tissue expansion, increased receptor-mediated clearance of natriuretic peptides in adipocytes, coupled with inherently diminished peptide secretion often observed in obesity, may further aggravate metabolic disturbances. Additionally, suboptimal natriuretic peptide levels could attenuate aldosterone suppression, potentially fostering related metabolic complications (43). Thus, the observed link between elevated NHHR and reduced NT-proBNP levels likely reflects an early, reciprocal interaction between metabolic dysregulation and the natriuretic peptide system—manifesting as either a protective adaptation, an initial suppressive response, or a consequence of unmeasured confounding. Further prospective studies and mechanistic investigations are needed to fully elucidate this relationship.

Our research has presented several important clinical implications. Firstly, in the general and hospital-based population devoid of prevalent CVD, the novel composite lipid index NHHR combines multiple lipid parameters, such as TC and LDL-C, allows for a comprehensive assessment of individual lipid status, offering the advantages of simplicity, ease of application, and cost-effectiveness. The consistent observation of an inverse association between NHHR and NT-proBNP across distinct populations presents a finding that diverges from conventional understanding. Specifically, the coexistence of a higher atherosclerotic lipid burden with lower circulating NT-proBNP levels suggests a potential bidirectional regulatory interaction between lipid metabolism and the natriuretic peptide system. Future research should prioritize external validation of this association across diverse and independent groups to further confirm its stability and generalizability. Notably, our finding represents the first to establish a connection between NHHR and NT-proBNP in a general and hospital-based population without CVD, employing independent research groups from different sources while adequately adjusting for potential confounding factors. This methodological rigor effectively captures the true epidemiological findings within the population.

We acknowledge certain methodological limitations. Due to the cross-sectional nature of this study, causal relationships and temporal order between NHHR and NT-proBNP concentrations cannot be definitively determined. In Group 1, the echocardiographic examination was not completed, precluding accurate estimation of the impact of cardiac function on serum NT-proBNP levels. Moreover, group 1 was derived from a nationwide probability sample representing the general population, Group 2 comprised a hospital-based inpatient cohort specifically selected for the absence of CVD. This fundamental difference in sampling frames inherently limits the generalizability of findings from Group 2 to the broader non-hospitalized population, as the hospital setting may introduce spectrum bias and restrict the clinical characteristics represented. Additionally, participant selection from the general population relied on self-reported CVD history, which may have introduced bias due to subjective reporting. Finally, Using a uniform NT-proBNP threshold to define elevated levels across both groups, despite their differences in age structure and disease spectrum, may introduce misclassification bias.

## Conclusion

In the general and hospital-based population, we observed a negative association between NHHR and NT-proBNP elevation, with an inflection point at 4.3 (Group 1). Below this threshold, NHHR exhibited a negative correlation with elevated NT-proBNP; however, beyond the inflection point, the relationship between the two variables became non-significant. These findings suggest that NHHR, as a straightforward composite lipid index, is associated with circulating levels of the cardiac biomarker NT-proBNP. Further longitudinal investigations are warranted to elucidate the temporal relationship and potential causal pathways linking lipid profiles to the dynamics of cardiac biomarkers.

## Data Availability

Publicly available datasets were analyzed in this study. The research encompassed the examination of publicly accessible datasets. The particular data employed is obtainable via the following URL: https://wwwn.cdc.gov/nchs/nhanes/Default.aspx.
