# Supplementary material for: Non-HDL-C/HDL-C Ratio and N-Terminal Pro-B-Type Natriuretic Peptide in the general and hospital-based population: a cross-sectional validation study
Source: Front Endocrinol (Lausanne). 2026 Jan 9;16:1739961. doi: 10.3389/fendo.2025.1739961 (PMC12827067; doi:10.3389/fendo.2025.1739961)
Supplement: Supplementary file 1 [file DataSheet1.docx]

**Non-HDL-C/HDL-C Ratio (NHHR) and N-Terminal Pro-B-Type Natriuretic Peptide in the general and hospital-based population: a cross-sectional validation study**

Haitao Xie^1,2,3^, Jianghong Li^3^, Le Shen^1,2^, Peng Yu^1,2^, Shi Wang^1,2^, Xiaohu Chen^1,2^, Shuhua Tang^1,2^

**Author Affiliations:**

1. Affiliated Hospital of Nanjing University of Chinese Medicine, Nanjing, China;
2. Jiangsu Province Hospital of Chinese Medicine, Nanjing, China;
3. Nanjing University of Chinese Medicine, Nanjing, China

**Corresponding author:**

Shuhua Tang, Jiangsu Province Hospital of Chinese Medicine, No. 155, Hanzhong road, Nanjing, China, 210004.

E-mail address: suewang10@163.com

Xiaohu Chen, Jiangsu Province Hospital of Chinese Medicine, No. 155, Hanzhong road, Nanjing, China, 210004.

E-mail address: chenxhdoctor@126.com

**Contact information:**

Haitao Xie: xht18356003693@163.com

Jianghong Li: [lijianghong2022@163.com](mailto:lijianghong2022@163.com)

Le Shen: [leshen1024@126.com](mailto:leshen1024@126.com)

Peng Yu: yupengdoctor@126.com

Shi Wang: suewang08@126.com

Xiaohu Chen: chenxhdoctor@126.com

Shuhua Tang: [suewang10@163.com](mailto:suewang10@163.com)

Table S1. Characteristics of hospital-based population by NHHR status, JSHTCM (Group 2).

| NHHR | Q1 | Q2 | Q3 | Q4 | p |
| --- | --- | --- | --- | --- | --- |
| n | 73 | 72 | 73 | 72 |  |
|  | < 2.181 | 2.181 - < 2.782 | 2.782 - < 3.331 | ≥ 3.331 |  |
| age | 64.77 (8.74) | 64.76 (8.92) | 61.73 (7.92) | 59.47 (9.82) | <0.001 |
| Gender (%) |  |  |  |  |  |
| male | 37 | 45.8 | 41.1 | 56.9 | 0.089 |
| female | 63 | 54.2 | 58.9 | 43.1 |  |
| Hypertension (%) |  |  |  |  |  |
| NO | 23.3 | 38.9 | 39.7 | 30.6 | 0.116 |
| Yes | 76.7 | 61.1 | 60.3 | 69.4 |  |
| Smoking (%) |  |  |  |  |  |
| Never | 78.1 | 75 | 82.2 | 65.3 | 0.266 |
| Current | 15.1 | 15.3 | 11 | 26.4 |  |
| Former | 6.8 | 9.7 | 6.8 | 8.3 |  |
| BMI | 24.63 (3.79) | 25.54 (3.05) | 24.86 (2.95) | 26.04 (3.71) | 0.053 |
| Urea (mmol/l) | 5.48 (1.30) | 5.69 (1.18) | 5.61 (1.59) | 5.76 (1.43) | 0.655 |
| Creatinine (umol/l) | 67.58 (13.45) | 70.11 (15.71) | 67.78 (19.12) | 71.73 (20.09) | 0.414 |
| Egfr (ml/min/1.73 m^2^) | 88.36 (13.30) | 87.34 (14.31) | 91.38 (13.82) | 91.30 (16.10) | 0.222 |
| TC (mmol/l) | 3.74 (0.83) | 4.11 (0.96) | 4.52 (0.97) | 5.07 (1.02) | <0.001 |
| HDL (mmol/l) | 1.33 (0.32) | 1.18 (0.26) | 1.11 (0.23) | 1.04 (0.19) | <0.001 |
| TG (mg/dl) | 98.32 (55.47) | 108.17 (41.08) | 144.34 (74.70) | 187.72 (100.08) | <0.001 |
| Uric acid (umol/l） | 302.74 (85.10) | 315.53 (72.30) | 304.73 (81.94) | 359.28 (99.53) | <0.001 |
| FBG (mg/dl) | 94.98 (19.71) | 97.27 (25.71) | 103.00 (23.96) | 97.28 (17.60) | 0.154 |
| HbA1c (%) | 6.09 (0.76) | 6.11 (0.92) | 6.29 (1.07) | 6.22 (0.96) | 0.541 |
| NT-proBNP, pg/mL | 117.47 (121.40) | 92.90 (93.38) | 76.25 (102.86) | 44.85 (45.79) | <0.001 |
| Elevated NT-proBNP (%) |  |  |  |  |  |
| No | 67.1 | 72.2 | 82.2 | 94.4 | <0.001 |
| Yes | 32.9 | 27.8 | 17.8 | 5.6 |  |
| Diabetes (%) |  |  |  |  |  |
| No | 84.9 | 76.4 | 72.6 | 81.9 | 0.26 |
| Yes | 15.1 | 23.6 | 27.4 | 18.1 |  |
| medication (%) |  |  |  |  |  |
| NO | 39.7 | 51.4 | 60.3 | 72.2 | 0.001 |
| Yes | 60.3 | 48.6 | 39.7 | 27.8 |  |

Table S2. Adjusted association of NHHR with NT-proBNP elevation in hospital-based population (Group 2).

| Association of NHHR with NT-proBNP elevation, Group 2 | | | | | | |
| --- | --- | --- | --- | --- | --- | --- |
| Outcome | Crude model |  | Model I |  | Model II |  |
|  | OR (95% CI) | p value | OR (95% CI) | p value | OR (95% CI) | p value |
| NHHR | 0.34 (0.22, 0.53) | < 0.001 | 0.40 (0.25, 0.64) | < 0.001 | 0.45 (0.25, 0.77) | 0.003 |
| NHHR (quartile) |  |  |  |  |  |  |
| Q1 | Reference |  | Reference |  | Reference |  |
| Q2 | 0.78 (0.39, 1.60) | 0.504 | 0.79 (0.37, 1.68) | 0.539 | 0.91 (0.40, 2.09) | 0.829 |
| Q3 | 0.46 (0.31, 0.99) | 0.047 | 0.57 (0.35, 1.31) | 0.191 | 0.73 (0.28, 1.88) | 0.521 |
| Q4 | 0.31 (0.13, 0.46) | < 0.001 | 0.36 (0.25, 0.53) | 0.002 | 0.42 (0.26, 0.80) | 0.021 |
| P for trend |  | < 0.001 |  | 0.001 |  | 0.035 |
| Association of NHHR with NT-proBNP elevation, Group 2 | | | | | | |
| Outcome | Crude model |  | Model I |  | Model II |  |
|  | β (95% CI) | p value | β (95% CI) | p value | β (95% CI) | p value |
| NHHR | -36.48 (-50.06, -22.91) | < 0.001 | -25.24 (-38.54, -11.94) | < 0.001 | -21.32 (-36.92, -5.73) | 0.007 |
| NHHR (quartile) |  |  |  |  |  |  |
| Q1 | Reference |  | Reference | < 0.001 | Reference |  |
| Q2 | -24.58 (-55.52, 6.37) | 0.121 | -22.76 (-51.93, 6.40) | 0.127 | -23.30 (-53.67, 7.08) | 0.134 |
| Q3 | -39.38 (-70.44, - 8.33) | 0.013 | -28.81 (-58.21, 0.581) | 0.055 | -21.74 (-53.43, 9.95) | 0.179 |
| Q4 | -73.54 (-94.27, -42.81) | < 0.001 | -50.63 (-80.43, -20.83) | < 0.001 | -41.90 (-76.16, -7.63) | 0.017 |
| P for trend |  | < 0.001 |  | 0.001 |  | 0.026 |
